# Supplementary material for: MSFragger-Labile: A Flexible Method to Improve Labile PTM Analysis in Proteomics
Source: Mol Cell Proteomics. 2023 Mar 31;22(5):100538. doi: 10.1016/j.mcpro.2023.100538 (PMC10182319; doi:10.1016/j.mcpro.2023.100538)
Supplement: Supplemental data [file mmc1.docx]

Supporting Information for

MSFragger-Labile: A Flexible Method to Improve Labile PTM Analysis in Proteomics

Daniel A. Polasky^1^, Daniel J. Geiszler^2^, Fengchao Yu^1^, Kai Li^2^, Guo Ci Teo^1^, Alexey I. Nesvizhskii^1,2^

^1^Department of Pathology, University of Michigan

^2^Department of Computational Medicine and Bioinformatics, University of Michigan

Contents

1. Figure S1. Example spectrum of a doubly phosphorylated peptide.
2. Figure S2. Zoomed-in view of the shaded region of the spectrum in Figure S1.
3. Figure S3. Example spectrum of a triply phosphorylated peptide.
4. Figure S4. Zoomed-in view of the shaded region from Figure S3.
5. Figure S5. Example spectrum of a doubly ADP-ribosylated peptide from AIETD
6. Figure S6. Example spectrum of a doubly ADP-ribosylated peptide from HCD
7. Figure S7. Zoomed-in view of the peptide backbone fragment region from Figure S6
8. Figure S8. Example spectrum of a peptide with ADP-ribose and another modification.
9. Figure S9. Comparison of HCD ADP-ribosylation search without diagnostic and peptide remainder ions – HeLa cell lysate data.
10. Figure S10. Comparison of HCD ADP-ribosylation search without diagnostic and peptide remainder ions – mouse liver tissue data.
11. Figure S11. Comparison of Martello et al. search results to MSFragger.
12. Table S1. PSM counts for Tran *et al.* phospho searches at each collision energy


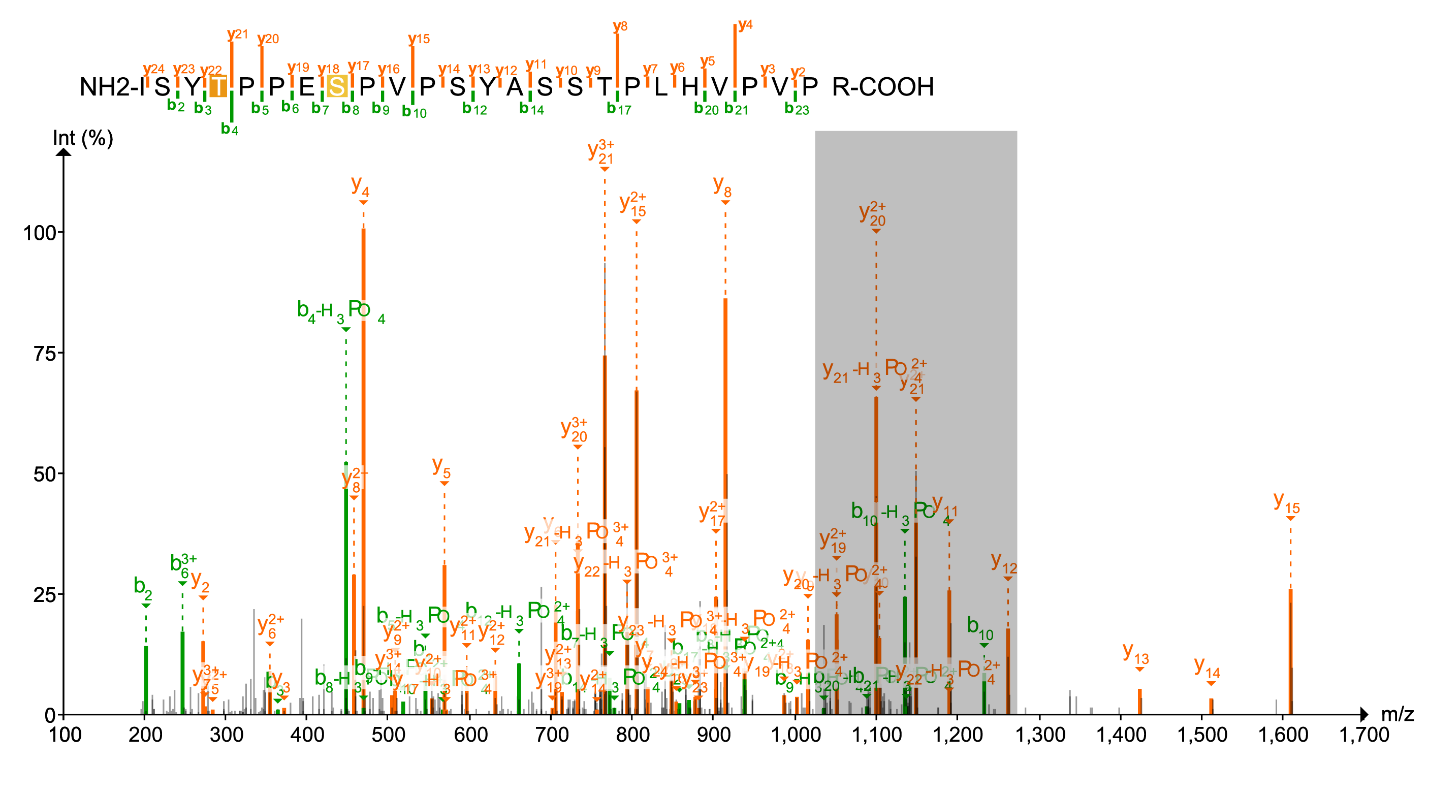


Figure S1. Example spectrum of a doubly phosphorylated peptide identified by labile search. Sites T4 and S8 (colored orange and yellow, respectively, on the sequence depiction at top) were identified as the phosphosites. Extensive phosphate neutral losses can be seen in the spectrum, including peaks with double phosphate loss (see Fig S2), confirming the double phosphorylation. Shaded region is expanded in Fig S2, below.


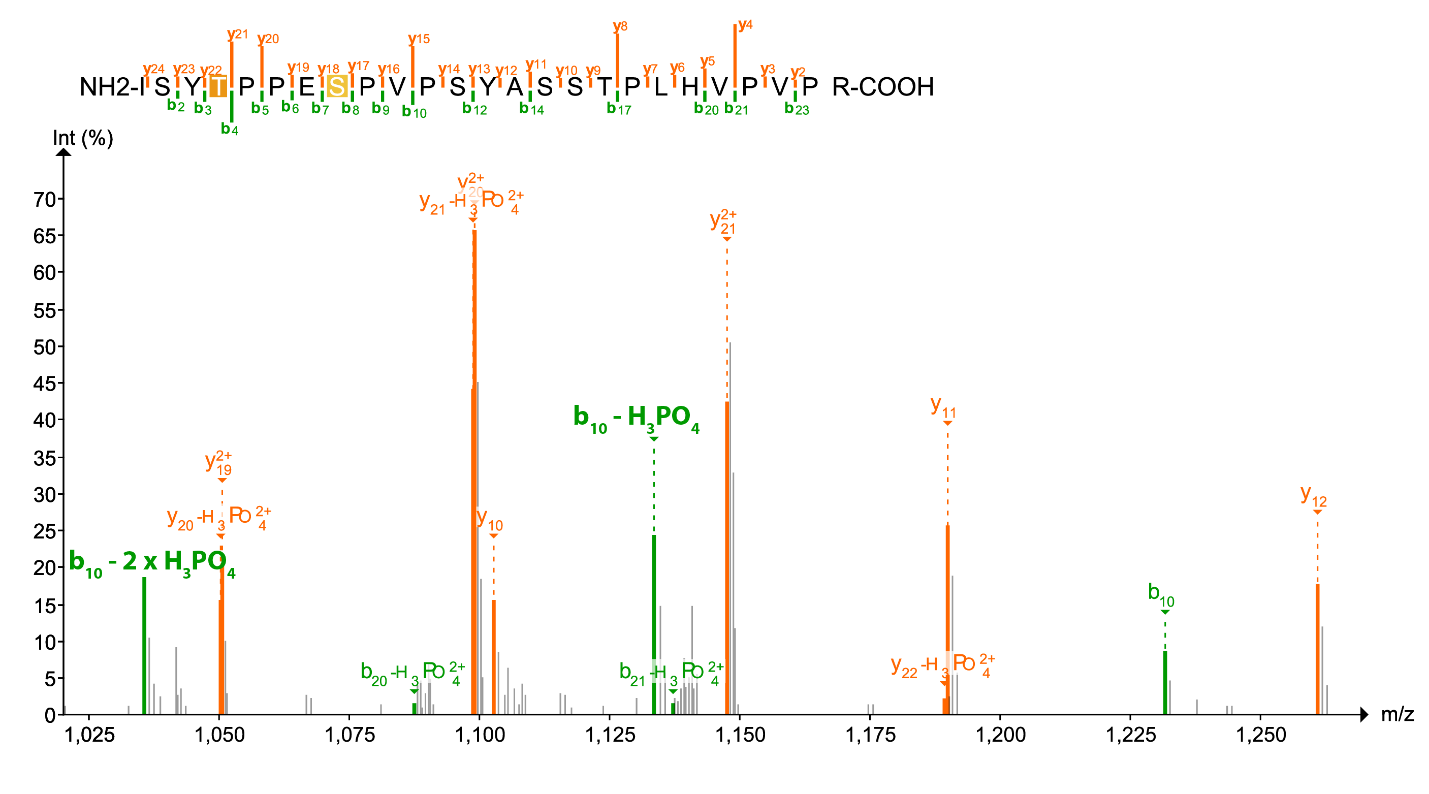


Figure S2. Zoomed-in view of the shaded region of the spectrum in Figure S1, showing the b_10_ ion with 0, 1, and 2 phosphate neutral losses (green, neutral losses in bold).


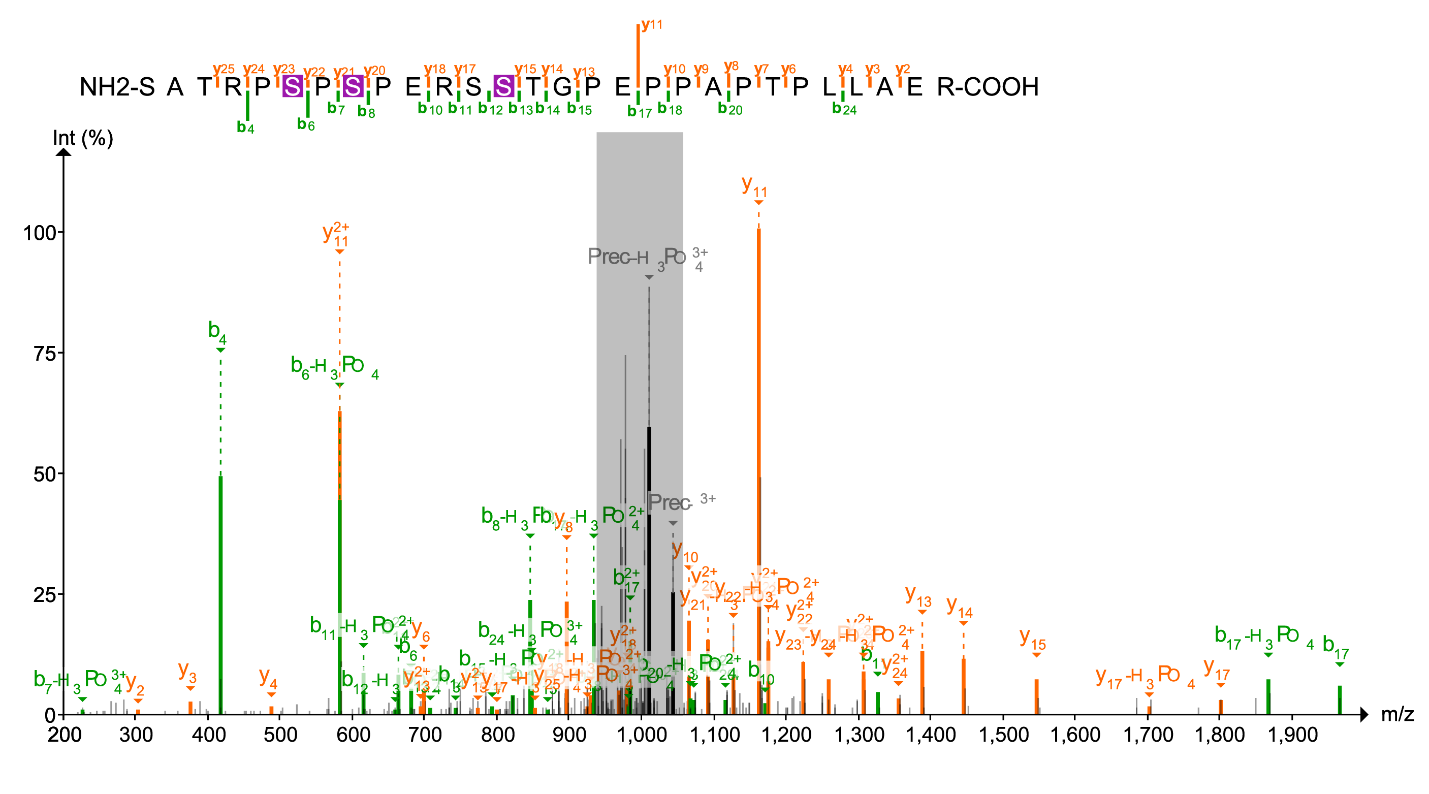


Figure S3. Example spectrum of a triply phosphorylated peptide identified by labile search. Sites S6, S8, and S13 were determined to be the most likely phosphosites by PTMProphet, though the probability of T14 phosphorylation is only slightly lower than S13, indicating possible ambiguity in the localization. Extensive phosphate neutral losses can be observed, including 3 distinct phosphate loss peaks from the precursor (see shaded region expanded in Fig. S4, below).


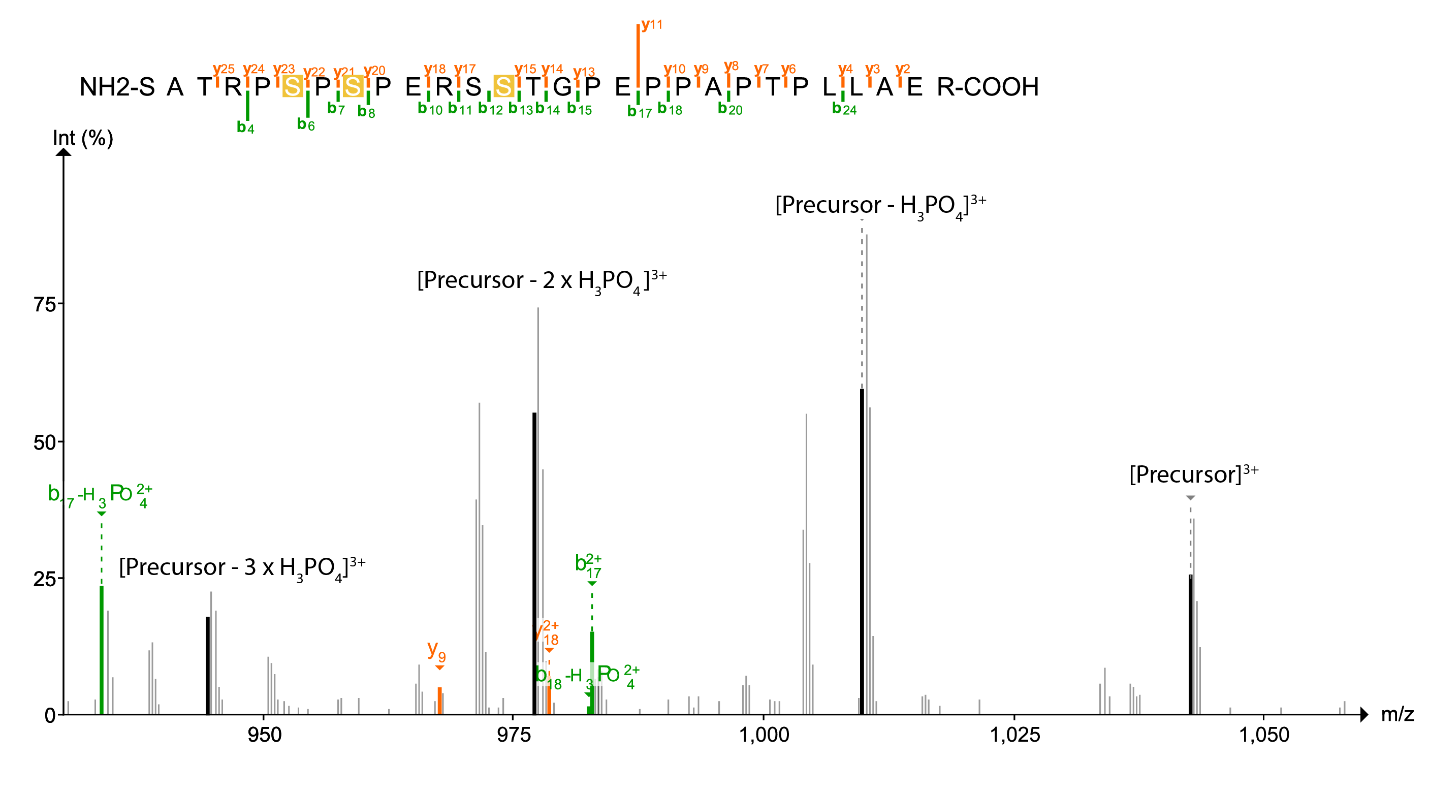


Figure S4. Zoomed-in view of the shaded region from Fig. S3, showing precursor and 3 successive losses of phosphate, indicating a triply phosphorylated peptide. The prominent unlabeled peaks to the left of each phosphate-loss peak result from an additional neutral loss of water.


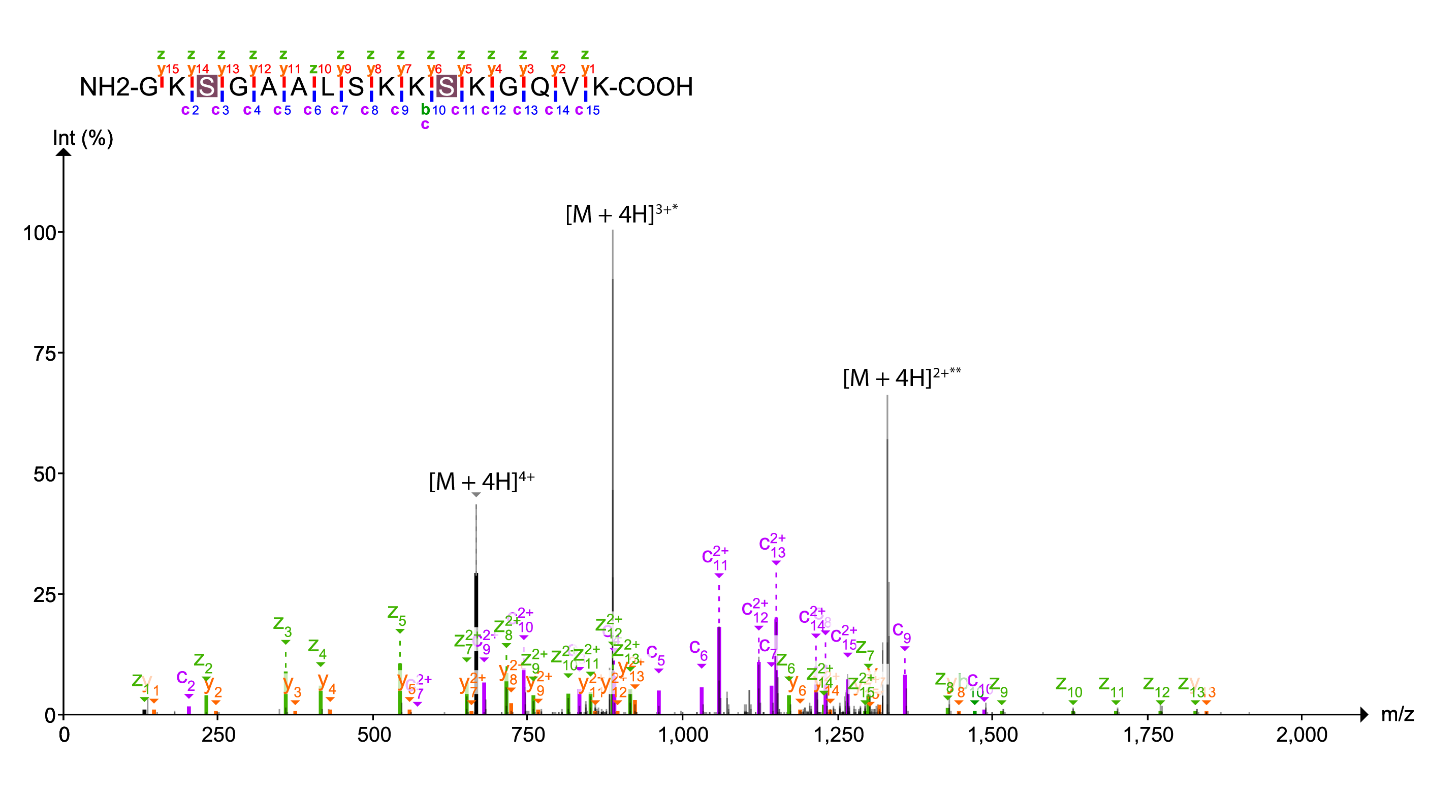


Figure S5. Example spectrum of a doubly modified ADP-ribosylated peptide detected with hybrid mode search. Sites S3 and S11 are ADP-ribosylated, as indicated by the c and z fragment ions bearing the intact modifications. AI-ETD activation with 15% laser power was used, hence the low proportion of b and y fragment ions compared to c and z and large electron transfer-no dissociation (ET-no-D) peaks.


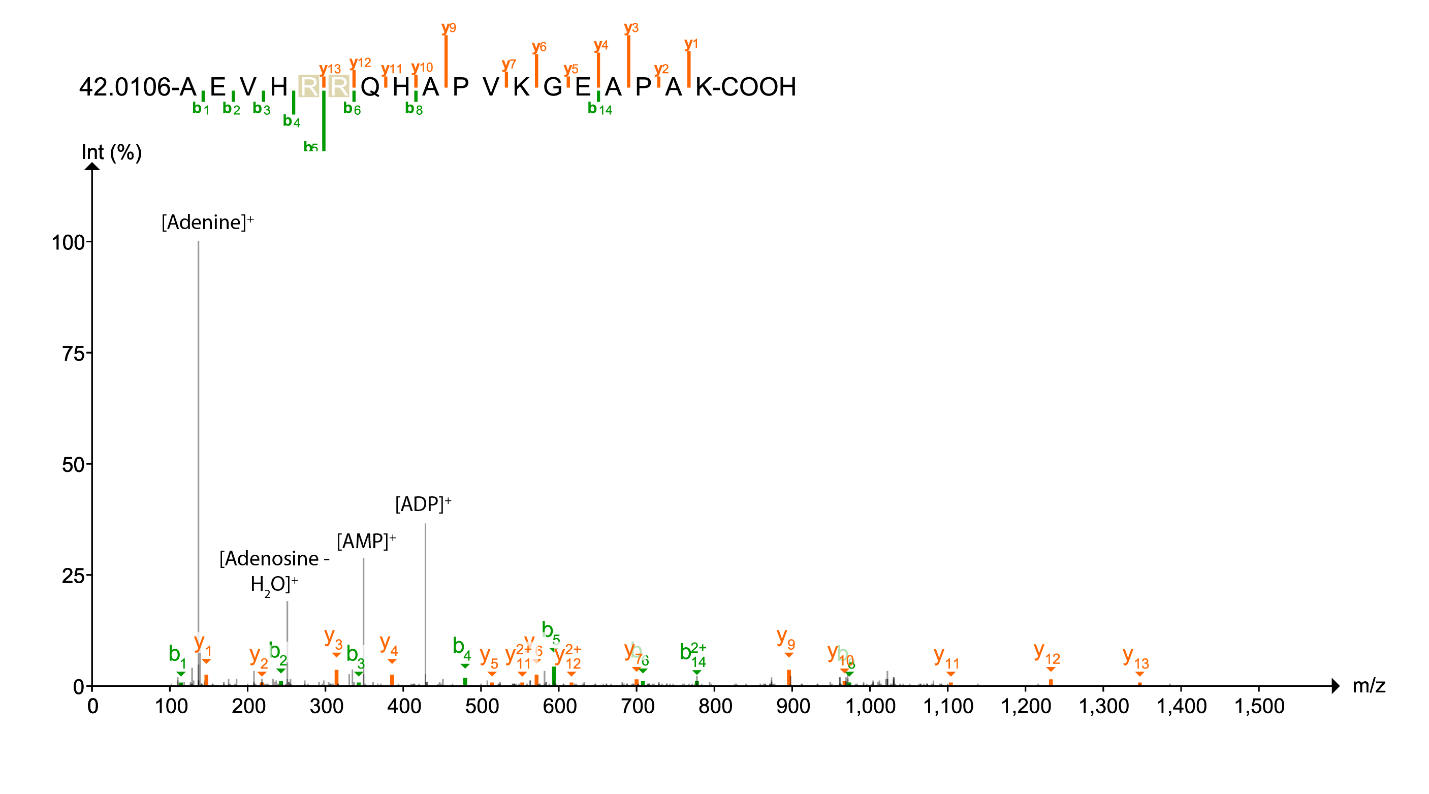


Figure S6. Example spectrum of a doubly modified ADP-ribosylated peptide from HCD data, annotated using fragment remainder ion (-42.0205) at each modified Arg. Several fragment ions containing the modification sites are found using this Arg-specific fragment remainder. No ions containing the modification site(s) are found using the intact modification (conventional search).


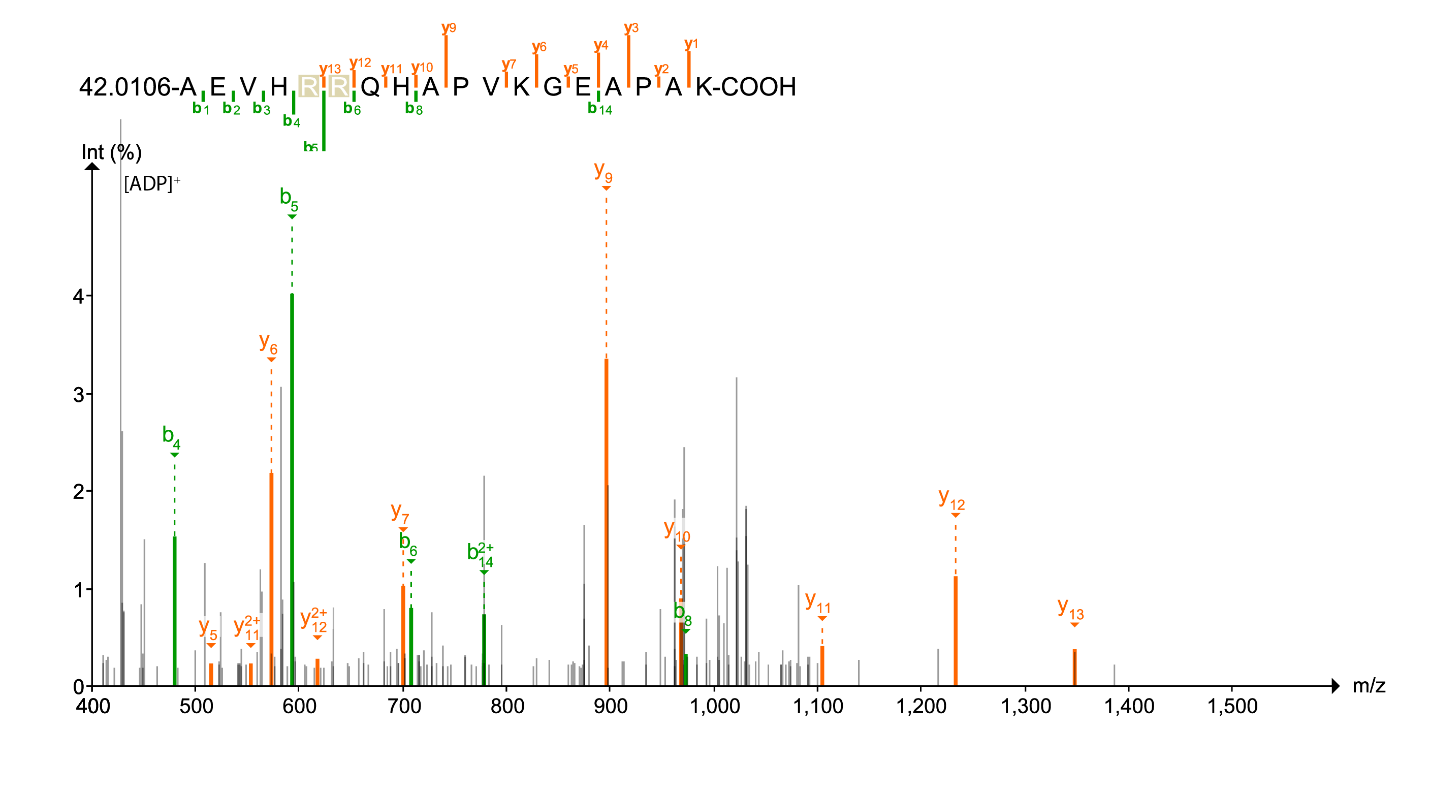


Figure S7. Zoomed view of the same example spectrum as in Fig. S6 with fragment ions annotated as the -42.0205 remainder fragment at each modified Arg.


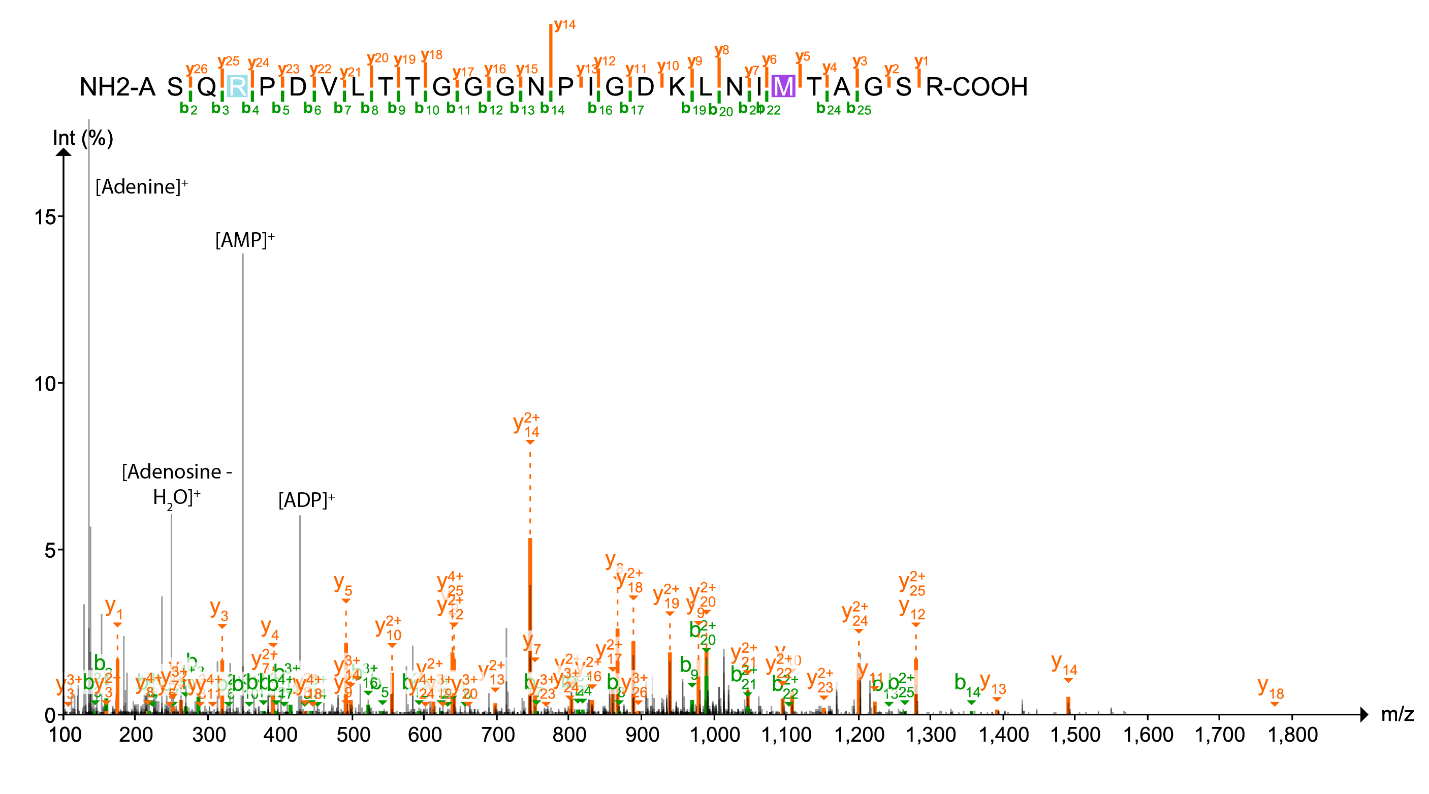


Figure S8. Example spectrum of a peptide with ADP-ribose and another (nonlabile) modification. The peptide has ADP-ribose at R4 at oxidation at M23. Fragment ions are annotated using the fragment remainder ion (-42.0205) at R4, corresponding to loss of ADP-ribose and part of the Arg side chain.

Figure S9. Comparison of HCD ADP-ribosylation search in Martello et al. HeLa cell data with and without diagnostic ions and peptide remainder ions. The “base” labile search (left) includes both diagnostic and peptide remainder ions. For both diagnostic and peptide remainder ions, removing them from the search results in a slight decrease in the number of ADP-ribose PSMs identified.

Figure S10. Comparison of HCD ADP-ribosylation search in Martello et al. mouse liver tissue data with and without diagnostic ions and peptide remainder ions. The “base” labile search (left) includes both diagnostic and peptide remainder ions. For this dataset, removal of diagnostic ions resulted in a slight decrease in the number of ADP-ribose PSMs identified, whereas removing peptide remainder ions actually increased the number of ADP-ribose PSMs. This may be because the peptide remainder ions are less abundant for ADP-ribose on Arg residues, which are more common in the mouse tissue than ADP-ribose on Ser, which was more common in the HeLa cell data.

Figure S11. Comparison of Martello *et al*. search results to MSFragger. The original results from Martello *et al.* used MaxQuant to search for ADP-ribosylation in HCD data, specifying it as a variable modification in a conventional search on K, R, D, and E residues. MSFragger searches specified K, R, D, E, and S residues as our analysis indicated that >80% of all ADP-ribose detected was likely present on Serine. At least part of the lower count of ADP-R PSMs in the original MaxQuant search is likely due to the fact that it did not allow ADP-ribose on S. MSFragger labile and hybrid searches each identified about 200 more ADP-R PSMs than MSFragger conventional search (as shown in Fig. 4).

Table S1. PSM counts for Tran *et al.* phospho searches at each collision energy. Number of offsets refers to the labeling in Fig. 3, with 0 offsets meaning nonlabile (conventional) search, 3 offsets meaning fully labile search, and 1 and 2 offsets meaning combination searches.

| Collision Energy | Number of Offsets | Total PSMs | Phospho PSMs |
| --- | --- | --- | --- |
| HCD-25 | 0 | 231241 | 119317 |
| HCD-25 | 1 | 242048 | 131369 |
| HCD-25 | 2 | 243469 | 133216 |
| HCD-25 | 3 | 243075 | 132498 |
| HCD-35 | 0 | 131423 | 72288 |
| HCD-35 | 1 | 140611 | 81733 |
| HCD-35 | 2 | 143683 | 84921 |
| HCD-35 | 3 | 150647 | 94722 |
